# Prevalence and clinical profile of metabolic syndrome in longevity: study from Guangxi Zhuang Autonomous Region, China

**DOI:** 10.1186/s12877-017-0536-y

**Published:** 2017-07-31

**Authors:** Xianghua He, Wei Zhang, Guofang Pang, Yuan Lv, Caiyou Hu, Ze Yang

**Affiliations:** 1 Department of Neurology, Jiangbin Hospital, No 85 Hedi Road, Nanning, Guangxi Zhuang Autonomous Region 530021 China; 2 The MOH Key Laboratory of Geriatrics, Beijing Hospital, National Center of Gerontology, No.1 Dahua Road, Dongdan, Beijing, 100730 People’s Republic of China

**Keywords:** Longevity, Metabolic syndrome, Chinese

## Abstract

**Background:**

Metabolic syndrome (MetS) was a risk factor for cardiovascular diseases, yet the prevalence of MetS among nonagenarians and centenarians was rarely reported. Here we investigated the prevalence of MetS and its components among nonagenarians and centenarians in our Zhuang population from Bama, Guangxi Zhuang Autonomous Region, China.

**Method:**

In Bama area, there registered 881 individuals who lived more than 90 years old in 269,800 local residents and our study involved 307 long-lived participants and 486 local younger (35–68 years) persons, as controls. MetS was defined according to the revised National Cholesterol Education Program’s Adult Treatment Panel III (NCEP ATPIII) criteria.

**Results:**

The overall prevalence estimates of MetS among longevity group were 28.0% based on NCEP ATPIII criteria. The most common metabolic component was elevated blood pressure (61.1%), followed by raised fasting glucose (39.1%) and low high-density lipoprotein cholesterol (low HDL-C) (28.0%). The prevalence of MetS and abdominal obesity in women (33.6% and 22.1% respectively) was higher than that of men (19.8% and 3.7% respectively) (P_range_ < .001–0.019). Compared with controls, long-lived individuals were more likely to have two or more metabolic abnormalities (P_range_ < 0.001), and less likely to have zero or one metabolic abnormality (P_range_ < 0.001–0.020).

**Conclusion:**

This study showed substantiality the prevalence and clinical profile of MetS in longevity population in Guangxi Zhuang Autonomous Region, China.

## Background

Individuals who live the longest are of broad interest to researchers in recent years. In 2010, there were 1,948,286 nonagenarians (people aged 90–99) and 35,934 centenarians (people aged 100 and more) in China [1]. As the population of China aging, the number of people aged 90 or more is expected to grow. Since persons who live into their 90s and over 100 are a testament to longevity, studies about their unique characteristics will expand our knowledge on how to extend life expectancy. Bama County is an autonomous county under the jurisdiction of Hechi City, Guangxi Zhuang Autonomous Region. It is located in the northwest of Guangxi Zhuang Autonomous Region with a total area of 1966 km^2^ and a population of 269,800 in 2010 [2]. Of which, 881 individuals have lived more than 90 years old [3].

Metabolic syndrome (MetS), a constellation of metabolic disorders including obesity, raised blood pressure, dyslipidemia (raised triglycerides and lowered high-density lipoprotein cholesterol), and raised fasting glucose, may be of special interest because of the increased prevalence with age [4, 5]. Studies about longevity population also revealed different types of metabolic disturbances [6–12], but that the results varied across different countries and ethnics. Study from Croatia suggesting that nonagenarians and centenarians had lower prevalence of overweight, obesity and lower blood pressure [6]. Instead, centenarians from Poland showed that mildly elevated blood pressure is a marker for better health status [7]. Recent study of familial longevity from China revealed decreased diastolic blood pressure but increased systolic blood pressure in centenarians [8]. Similar discrepancy can also be found among studies of lipid profile and longevity. Biological study for longevity demonstrated that centenarians and their offspring have significantly larger high-density lipoprotein (HDL) levels and particle sizes and low-density lipoprotein (LDL) levels compared with controls [9, 10]. However, other studies did not find significant association of HDL-C levels with centenarians [11, 12].

All of these studies indicated that individuals with longevity might have different metabolic phenotypes from those general individuals under different ethnic background. But no reports on these metabolic items research integrated were seen now yet. Furthermore, the prevalence of MetS increased in Chinese population aged 60–95 [13]. Thus we perform the study to investigate the prevalence and clinical profile of MetS in longevity in Guangxi Zhuang Autonomous Region, China.

## Methods

### Study population

The project is a cross-sectional study within the framework of the “Longevity and Health of Aging Population in Guangxi China (LHAPGC)” [14]. In this study, “longevity” subjects were classified as participants who had survived to age 90 years or more, with “unrelated younger controls” aged 35–68 years. A random sample of 793 individuals belonged to the Zhuang population from Bama (total population: 269,800) was recruited, including 307 long-lived individuals (256 nonagenarians and 51 centenarians) and 486 local and unrelated younger controls. Zhuang population is one of the largest ethnic groups in mainland China, second only to the Han population. The individuals with longevity included 226 women and 81 men (mean age: 95.06 ± 4.91 years and 94.60 ± 4.09 years old for women and men, respectively; range: 90–111 years old). The control group comprised 185 women and 301 men (mean age: 47.98 ± 4.07 and 47.24 ± 3.70 years old for women and men, respectively; range: 35–68 years old).

The survey was conducted using a uniform standardized protocol. All participants were examined by a senior physician and underwent extensive neuropsychological test as well as taking instrumental examination such as electrocardiogram and ultrasound examination. Individuals with longevity as well as controls were excluded if they had chronic disease such as malnutrition, hepatic disease, kidney disease and cancer. All controls refer to local and unrelated younger participants in general population.

The study was conducted according to the principles expressed in the Declaration of Helsinki. The Ethics Committee of Beijing Hospital, Ministry of Health approved the study protocol. Written informed consent was obtained from each of the participants.

### Measurements

MetS was diagnosed as three or more of the following five factors as defined by the revised National Cholesterol Education Program’s Adult Treatment Panel III (NCEP ATPIII) criteria for Asians (the American Heart Association and the National Heart, Lung, and Blood Institute (AHA/NHLBI) revised in 2005 [4], the same as the joint interim statement in 2009 [5]: (1) waist circumference ≥ 90 cm in males and ≥80 cm in females; (2) systolic blood pressure (SBP) ≥ 130 mmHg or diastolic blood pressure (DBP) ≥ 85 mmHg or taking anti-hypertensive drugs; (3) fasting blood glucose ≥ 5.6 mmol/L or taking drugs for diabetes; (4) triglycerides ≥ 1.7 mmol/L or taking antihyperlipidemic drugs; (5) HDL-C < 1.03 mmol/L in males and <1.29 mmol/L in females or taking antihyperlipidemic drugs.

Body mass index (BMI) was determined as weight (kg) divided by height (m) squared. Waist circumference was measured at the mid-point between the lowermost rib and the iliac crest.

### Laboratory measurements

Blood samples were collected after at least eight hours overnight fast for serum biochemistry, lipid profile, and plasma glucose in all participants. Clinical biomarkers including fasting plasma glucose (FPG), triglyceride (TG), and high-density lipoprotein cholesterol (HDL-C) were determined following standard laboratory procedures. Blood pressure was measured using a standard mercury sphygmomanometer on the right arm after at least 10 min of rest.

### Statistical analysis

All statistical analyses were conducted using the SPSS 18.0 software package. One-Way ANOVA and a chi-squared test were used to compare demographic and clinical data between longevity and controls. Age- and sex- adjusted were applied according to the 2010 population census of the people’s republic of China from National Bureau of Statistics [1]. A two-tailed *P* < 0.05 was considered statistically significant.

## Results

### Demographic and metabolic characteristics in two groups

Data for demographic characteristics were showed as numbers, and data for metabolic characteristics were presented as median and interquartile range (INR) in Table 1. Except for frequency of alcohol intake (*P* = 0.772), there were significant difference for frequency of cardiovascular disease, smoking status and sex ratio between the longevity group and the control one (P_range_ < .001). The study consisted of 793 participants with a female preponderance in the longevity group (Male: Female =1:2.79). Generally, subjects in the long-lived individuals cohort had significantly lower levels of height, body mass index (BMI), weight, waist circumference (WC), and TG than controls (P_range_ < .001–0.002), except for systolic blood pressure (SBP), diastolic blood pressure (DBP), and FPG (P_range_ < .001) (see Table 1). There was no difference of HDL-C level between two groups (*P* = 0.766).

**Table 1:** Demographic and metabolic characteristics of the study samples between longevity and controls

Variables	Longevity(*N* = 307)	Control(*N* = 486)	*P*
Cardiovascular disease(N)	17	0	<.001
Smoking status(N)			
Never	295	420	<.001
Former	12	15	
Current	0	51	
Cigarettes per day (current)(N)			
≤ 10	0	35	-
≥ 11	0	31	
Frequency of alcohol intake(N)			
1–3 times/month	6	10	0.772
1–2 times/week	23	44	
3–4 times/week	8	20	
Nearly 1 time/day	5	6	
Drugs(N)			
Antidyslipidemic	0	0	-
Antihypertensive	2	0	
Antidiabetic	0	0	
Gender(M/F)(N)	81/226	301/185	<.001
Age (Median(INR))	94(91–98)	48(45–49)	<.001
Height (Median(INR))	145.0(140.0–151.0)	164.5(158.0–169.5)	<.001
Weight (Median(INR))	38(34–45)	66(58–72)	<.001
BMI (Median(INR))	18.35(16.41–20.93)	24.11(22.57–26.00)	<.001
SBP (Median(INR))	146(132–160)	120(110–130)	<.001
DBP (Median(INR))	80(72–90)	76(70–84)	<.001
WC (Median(INR))	71.0(65.0–78.0)	80.5(75.0–86.0)	<.001
FPG (Median(INR))	5.08(4.30–6.16)	4.89(4.47–5.24)	<.001
TG (Median(INR))	1.08(0.77–1.55)	1.52(1.22–1.86)	0.002
HDL-C (Median(INR))	1.40(1.10–1.81)	1.46(1.30–1.68)	0.766

*INR* interquartile range, *BMI* body mass index, *SBP* systolic blood pressure, *DBP* diastolic blood pressure, *WC* waist circumference, *FPG* fasting plasma glucose, *TG* triglyceride, *HDL-C* high-density lipoprotein cholesterol

### Overall prevalence of metabolic profile

Table 2 showed the prevalence of MetS among the longevity of Zhuang Population from Guangxi Zhuang Autonomous Region. The prevalence of MetS was significantly higher in the longevity group (28.0%) than it is in the local general control group (5.1%) (0R:0.139, 95%CI: 0.087–0.224, *P* < .001) regardless of the used criteria (P_range_ < .001) (see Table 2). The most common metabolic component in longevity individuals was high blood pressure (61.1%), followed by raised fasting glucose (39.1%) and lowHDL-C (28.0%). In addition, the prevalence of high blood pressure (HBP) (0R:0.106, 95%CI: 0.075–0.150, *P* < .001), raised fasting glucose (0R:0.121, 95%CI: 0.080–0.183, *P* < .001), and lowHDL-C (0R:0.241, 95%CI: 0.166–0.350, *P* < .001) was significantly higher in long-lived individuals than it is in control subjects. On the contrary, the prevalence of highTG (0R:1.980, 95%CI: 1.426–2.749, *P* < .001) was much lower in people with longevity than the controls (see Table 2). There was no difference of the abdominal obesity between longevity individuals and controls (0R:0.753, 95%CI: 0.508–1.116, *P* = 0.157) (see Table 2).

**Table 2:** Clinical metabolic characteristics between longevity and controls

	Longevity	Control			95%CI		
	*n* = 307(100%)	*n* = 486 (100%)	χ^2^	OR	Lower	Upper	*P*
AOB	17.3%	13.6%	2.002	0.753	0.508	1.116	0.157
Elevated BP	61.1%	38.9%	183.15	0.106	0.075	0.150	<.001
RFG	39.1%	7.2%	121.65	0.121	0.080	0.183	<.001
highTG	21.8%	35.6%	16.910	1.980	1.426	2.749	<.001
lowHDL-C	33.2%	10.7%	61.005	0.241	0.166	0.350	<.001
MetS	28.0%	5.1%	81.740	0.139	0.087	0.224	<.001

*95%CI* 95% Confidence Interval, *AOB* abdominal obesity, *Elevated BP* elevated blood pressure, *RFG* raised fasting glucose, *highTG* high triglyceride, *low HDL-C* low high-density lipoprotein cholesterol, *MetS* metabolic syndrome

### Prevalence of metabolic profile in longevity

Since numbers of male and female in longevity group are not equal, we compared the prevalence of clinical metabolic data among male and female participants. We did not find significant difference of age, height, weight, BMI, SBP, DBP, WC, FPG, TG and HDL-C among male and female long-lived individuals (P_range_ > 0.05).

Compared to males, the estimated odds of having MetS were 2.057 times higher in female longevity subjects (0R: 2.057, 95%CI: 1.118–3.787, *P* = 0.019) (see Table 3). At the same time, females were more likely to have abdominal obesity compared to males (*P* < .001) (see Table 3). No significant difference of prevalence for elevated BP, raised fasting glucose, highTG, and lowHDL-C among male and female individuals aged 90+ years were noted in this study (see Table 3).

**Table 3:** MetS and its components in longevity individuals stratified by gender

	Male	Female			95%CI		
	*N* = 81(100%)	*N* = 226(100%)	χ2	OR	lower	upper	*P*
AOB	3.70	22.10	-	7.386	2.236	24.405	<.001*
Elevated BP	79.00	83.60	0.876	1.357	0.715	2.574	0.349
RFG	43.20	37.60	0.785	0.792	0.473	1.327	0.376
highTG	18.50	23.00	0.705	1.315	0.693	2.495	0.401
lowHDL-C	27.20	35.00	1.641	1.441	0.823	2.525	0.200
MetS	19.80	33.60	5.507	2.057	1.118	3.787	0.019

^*^: *P* value according to Fisher’s Exact Test

*95%CI* 95% Confidence Interval, *AOB* abdominal obesity, *Elevated BP* elevated blood pressure, *RFG* raised fasting glucose, *highTG* high triglyceride, *low HDL-C* low high-density lipoprotein cholesterol, *MetS* metabolic syndrome

### Trend for frequency of metabolic abnormalities in two groups

In this study, people aged 90+ have much higher prevalence of MetS than that of the controls (28.0% vs. 5.1%) (See Table 2). For further analyzing the trend of MetS, we researched the prevalence of having zero, one, two, three, four, and five MetS components in the two groups. Table 4 showed that Compared with controls, long-lived individuals were more likely to have two or more components of MetS (for longevity: 36.2% and 28.0% respectively; for controls: 21.2% and 5.1% respectively; P_range_ < 0.001), and less likely to have zero or one components of MetS (for longevity: 6.2% and 29.6% respectively; for controls: 32.1% and 41.6% respectively; P_range_ < 0.001–0.020).

**Table 4:** Frequency of metabolic abnormalities in two groups

	Longevity			Control				
MetS components (N)	Frequency	Percent	Mean ± Sd.	Frequency	Percent	Mean ± Sd.	*P*	OR
0	19	6.2	95.21 ± 4.662	156	32.1	47.05 ± 4.030	<.001	0.117–0.317
1	91	29.6	95.21 ± 4.682	202	41.6	47.53 ± 3.341	0.02	0.536–0.949
2	111	36.2	94.88 ± 4.810	103	21.2	47.79 ± 4.144	0.001	1.259–2.232
3	63	20.5	94.63 ± 4.867	22	4.5	49.36 ± 5.141	<.001	2.733–7.519
4	19	6.2	94.68 ± 4.372	3	0.6	48.00 ± 4.359	<.001*	2.942–34.165
5	4	1.3	95.25 ± 2.986	0	0	-	0.023*	-
Total	307	100	94.94 ± 4.702	486	100	47.52 ± 3.859	-	-

^*^ *P* value according to Fisher’s Exact Test

*Std* standard deviation

## Discussion

The present study provided the information on the overall prevalence estimates of MetS in longevity group been 28.0% based on NCEP ATPIII criteria. The most common metabolic component was high blood pressure, followed by raised fasting glucose and lowHDL-C. The prevalence of MetS and abdominal obesity in women was higher than that of men. No significant difference of metabolic components among longevity participants were found after stratified by gender. The prevalence of MetS among longevity individuals was significantly higher than the local general control individuals. Interestingly, we found that compared with controls, longevous individuals were more likely to have two or more components of MetS, and less likely to have zero or one components of MetS.

To the best of our knowledge, rare studies examined the prevalence of MetS in longevity subjects. Studies from Sichuan Province, China, reported a prevalence rate of MetS with 9.3% to 10.8% in the total participants among individuals aged 90+ years old [15, 16], which was lower than ours. However, another Chinese cross-sectional study reported the prevalence rate of MetS was 50.4% in 2001 and 58.1% in 2010 among subjects aged 60–95 years [13], which was much higher than ours. The difference prevalence rate of MetS may be due to different ethnics and different human age stages. Further multicenter large-scale sample for longevity are needed to address this problem.

The most common metabolic component among longevity subjects in our Zhuang population was inconsistent with studies from other ethnic population [17, 18]. For example, studies performed in Korea found that the most common component was high blood sugar levels, followed by elevated triglyceride levels and high blood pressure in males, and that elevated triglyceride levels, followed by high blood sugar levels and high blood pressure in females in a 66-year-old population [18]. In China, it has been shown that the most common component was elevated blood pressure, followed by central obesity and raised fasting glucose among Chinese aged 60 years or older[19]. Since the age for the participants in these studies were much younger than ours, it is possible that these inconsistent findings regarding different prevalence of metabolic components could be age differences. Another possible explanation may be explained by ethnic differences.

Females with longevity had higher prevalence of MetS than the male participants, which were consistent with several studies from different countries and ethnic population [13, 17, 18]. It is interesting that women had higher prevalence rates of MetS compared with men. According to the National Bureau of Statistics of China, the female/male ratio of longevity population were 2.02:1 (648,588 male nonagenarians, 1,299,698 female nonagenarians, 8852 male centenarians and 27,082 female centenarians in 2010) [1]. In our study, females also occupied a larger proportion (female/male: 2.79/1) in the longevity population. Thus, this group may be more representative of an ordinary group for longevious people, and they may be more sensitive to the risk factors from MetS than men. In addition, women who become postmenopausal had a significantly increased visceral abdominal fat [20, 21], accompanied by insulin resistance and hypertriglyceridemia, ultimately meeting the diagnosis of metabolic syndrome[22]. However, for females aged 90+ years and for who had menopause for more than 40 years, it is hard to say hormonal regulation had an effect on their metabolic state. Additional consideration is required for sex differences between men and women among the oldest old.

In this study, women were more likely to have abdominal obesity than men in the longevity group. No significant difference of prevalence for high BP, dysglycemia, highTG, and lowHDL-C among male and female individuals aged 90+ years were observed in this study. This result was inconsistent with other studies. For example, studies performed in China shown that the prevalence of TG, HDL-C, and WC among females is higher than the prevalence in males among Chinese aged 60–95 years old [19]. In Turkey, researchers found that older adult females had higher SBP, larger WC, and lowHDL-C than older males [23]. In Korea, prevalence of abdominal obesity and lowHDL-C in females were higher than males, while prevalence of highTG, high BP, and dysglycemia in males were higher than females [18]. The difference among different countries may be due to different ethnics and sample size. Multicenter Collaboration across different countries is needed to address this question.

In our study, the high prevalence of MetS in longevity group could be linked to the observed higher prevalence of high BP, raised fasting glucose and lowHDL-C and lower prevalence of highTG compared to the control group. These findings were supported by previous studies which demonstrated nonagenarians and centenarians had higher prevalence of confirmed hypertension, diabetes mellitus as well as dyslipidemia [6–8, 24]. Moreover, a study investigating frailty and metabolic syndrome in a Chinese community sample showed that, in those aged 90 years and older, frailty was a significant risk for near-term death, regardless of the metabolic syndrome [16]. Furthermore, another cross-sectional study implicated that metabolic syndrome may be associated with better cognitive function among nonagenarians and centenarians [15]. Thus, it seemed that MetS was not a risk factor for the oldest old. However, it is well established that MetS is strongly related to increased incidence of cardiovascular events in people aged 60–95 [13, 25]. One possible explanation of why MetS has a different effect among different stages of human beings might be because of “natural selection effect.” It is possible that those with more severe MetS have died of cardiovascular diseases before reaching an older age. Another possible explanation is that MetS components are associated with better health status among the oldest old [7, 9, 26]. Specifically, low prevalence of highTG might contribute a lot to longevity in our study, as low TG level has been identified as a marker for human longevity [26, 27]. Lastly, there was a possibility that traditional cardiovascular risk factors, such as an elevated cholesterol and hypertension, might do not automatically apply to the very old.

Moreover, we found that long-lived individuals had higher frequency for having two or more metabolic abnormalities and lower frequency for having zero or one metabolic abnormality than the controls, which was consistent with previous studies that prevalence of MetS increased with age [13, 28]. More research is needed for the oldest old with MetS.

In our study, the control individuals had higher frequency of smoking than the longevity participants, which might explain the possibility for fewer controls having MetS, as smoking is a risk factor for younger people [29, 30].

However, our study has some limitations. Firstly, this was a cross-sectional study. Many variables were measured at a single time point and may be subject to conditions at the time of measurement. Since some of the study population had several risk factors including hyperlipidemia, we could not eliminate the possible effect of underlying diseases and medications used for these diseases. Secondly, the data came from a single region, which may limit generalizability. Thirdly, the sample size was not big enough in our study. Fourthly, the prevalence of various lipid and hematological parameters was based on a single assessment of blood, which may introduce a misclassification bias. Lastly, we have excluded Individuals with chronic disease such as malnutrition, hepatic disease, kidney disease and cancer, which might underestimate the prevalence of MetS in our population, especially in long-lived individuals. Multicenter collaboration in prospective research of prevalence for MetS in longevity group is needed to address these questions.

## Conclusions

In conclusion, our study analyzed the prevalence of MetS and its components distribution in longevity individuals in Zhuang population. We also reported that there existed the markedly differences of MetS rates by sex-specific groups in the population, and that long-lived individuals were more likely to have two or more metabolic abnormalities, and less likely to have zero or one metabolic abnormality than the controls.

## Abbreviations

95%CI: 95% Confidence Interval
AHA/NHLBI: American Heart Association and the National Heart, Lung, and Blood Institute
AOB: Abdominal obesity
BMI: Body mass index
DBP: Diastolic blood pressure
FPG: Fasting plasma glucose
HDL-C: High-density lipoprotein cholesterol
INR: Interquartile range
LDL-C: Low-density lipoprotein cholesterol
MetS: Metabolic syndrome
NCEP ATPIII: National Cholesterol Education Program’s Adult Treatment Panel III
SBP: Systolic blood pressure
Sd: Standard deviation
TG: Triglyceride
WC: Waist circumference
